# The presence of tumour-infiltrating lymphocytes (TILs) and the ratios between different subsets serve as prognostic factors in advanced hypopharyngeal squamous cell carcinoma

**DOI:** 10.1186/s12885-020-07234-0

**Published:** 2020-08-05

**Authors:** Jie Wang, Shu Tian, Ji Sun, Jiahao Zhang, Lan Lin, Chunyan Hu

**Affiliations:** 1 Department of Radiotherapy, Eye & ENT Hospital, Fudan University, Shanghai, China; 2 Department of Radiotherapy, Eye & ENT Hospital, Fudan University, Shanghai, China; 3 Department of Pathology, Eye & ENT Hospital, Fudan University, 2600 jiangyue Road, Shanghai, 201112 China

**Keywords:** Tumour-infiltrating lymphocytes, CD8/Foxp3 ratio, CD8/CD4 ratio, Immunohistochemistry, Advanced hypopharyngeal squamous cell carcinoma

## Abstract

**Background:**

Cancer cells induce the infiltration of various immune cells that are located or distributed in different sites and play multiple roles, which have recently been proposed to predict clinical outcomes. We therefore studied the prognostic significance of the presence of tumour-infiltrating lymphocytes (TILs) and the ratios between different types of immune cells in hypopharyngeal squamous cell carcinoma (HPSCC).

**Methods:**

We retrospectively analysed 132 consecutive patients diagnosed with advanced HPSCC in 2013–2017. Tumoural parenchyma was immunohistochemically counted manually for the number of CD8, CD4 and Foxp3 cells. The ratios of CD8/Foxp3 and CD8/CD4 ratios were calculated for each specimen and analyzed with respect to patient clinicopathological variables and prognosis.

**Results:**

HPSCC patients with high levels of TILs showed evident correlations with well differentiated tumors (*P* < 0.05). Moreover, Foxp3+ TIL is also associated with overall staging group and T category (*P* = 0.048 and *P* = 0.046, respectively). Kaplan-Meier analysis showed that high CD8 and FoxP3 infiltration correlated with favourable overall survival (OS, *P* = 0.019 and *P* = 0.001), disease-free survival (DFS, *P* = 0.045 and *P* = 0.028) and distant metastasis-free survival (DMFS, *P* = 0.034 and *P* = 0.009), respectively, but only Foxp3 displayed prognostic significance for DMFS in multivariate analysis (MVA). In the lymphocyte ratio analysis, CD8/Foxp3 appeared to play a pivotal role, and patients with a high CD8/Foxp3 ratio had a superior 3-year DFS and DMFS compared with those a low CD8/Foxp3 ratio in both univariate analysis (UVA) and MVA (*P* = 0.015 and *P* = 0.011). A high CD8/CD4 ratio was associated with better DFS and local relapse-free survival (LRFS) in UVA, and was an independent prognostic factor for improved LRFS in MVA (*P* = 0.040).

**Conclusion:**

Although high TILs levels were determined to be prognostically significant in advanced HPSCC, the ratios of these subsets may be more informative. Particularly, a higher ratio of CD8/Foxp3 accurately predicts prognosis for improved DFS and DMFS, and an increased CD8/CD4 ratio is an independent predictor for favourable LRFS.

## Background

Hypopharyngeal squamous cell carcinoma (HPSCC) is a highly malignant type of head and neck cancer, which is the eighth most common cancer worldwide [1]. Although its incidence is comparatively low, HPSCC is usually diagnosed at an advanced stage due to unapparent early symptoms [2] . Although there are many treatments, such as surgery, concurrent chemoradiation therapy (CCRT) and radiation therapy, the five-year survival rate is less than 35% [3, 4]. Given the difficulty of diagnosing HPSCC at an early stage as well as its severe prognosis, new approaches concerning prognostic evaluation and treatment alternatives are necessary. It is urgent to find novel biological factors that accurately predict clinical outcomes for HPSCC patients.

In recent years, it has been increasingly recognized that the immune microenvironment is the “battlefield” between tumour progression and the immune system defence. Immune surveillance and immune escape provide a dynamic balance, inhibiting tumour progression by recognizing and killing tumour cells, and weakening the antitumour activity of immune cells by expressing inhibitory molecules and secreting cytokines [5]. Tumour-infiltrating lymphocytes (TILs), which are heterogeneous lymphocyte population mainly composed of T lymphocytes, are important in the tumour immune microenvironment; they were first proposed in 1986 and have been proven to be an independent prognostic biomarker in various tumours [6–9]. Growing evidence indicates that TILs consist of numerous antitumour effector or regulatory T cells (Tregs) and are key players in the host’s immune response to tumour. Thus, evaluating the functions of different TIL subsets may provide a better understanding of tumour progression and effective antitumour strategies. In fact, the most consistently beneficial TILs seem to be CD8+ TILs, which are regarded as cytotoxic T lymphocytes (CTLs), and specifically recognize and destroy target cells [10]. These cells have been reported to be the major effector cell for tumour elimination by recognizing tumour-derived antigenic epitopes [11]. In contrast, Foxp3+ TILs have been classified as Tregs, and may actually contribute to suppressing antitumour immune responses [12]. In most studies, Tregs are generally considered to play a crucial role in the process of immune escape, helping tumour cells avoid immunological surveillance. However, the prognostic significance of Foxp3+ TILs remains controversial. For instance, Foxp3+ TILs were reported to be linked to favourable clinical outcomes in non-small cell lung cancer (NSCLC) and sinonasal squamous cell carcinoma [13, 14], but others reported that Foxp3+ TILs were correlated with to worse prognosis [15, 16]. Furthermore, CD4+ TILs are derived from T cells mediated by IL-2, which include a T helper cell population and Tregs. In terms of antitumour immunity, T helper cell activation is effective and plays an important role in inducing or motivating CTLs, whereas CD4+ Tregs suppress effector T lymphocytes [17, 18]. However, whether these pro-tumour effects outweigh antitumour effects or are equal in a particular tumour is debatable. This could explain why the benefits of CD4+ T cell infiltration on the prognosis of different tumours are somewhat inconsistent. From the above, it is evident that TILs may act as a double-edged sword, and the relations between the different types of immune cells have not been thoroughly examined. More recently, the hypothesis that lymphocyte ratios could have more prognostic significance has gained much attention. Emerging evidence has shown that higher ratios of CD8+/Foxp3+ and CD8/CD4 are more sensitive indicators of prognosis and for monitoring immune function, even serving as biomarkers to predict tumour relapse and responses to treatment [13, 19–21]. A study by Sideras et al. examined the fresh metastatic tissues of 47 patients with colorectal cancer liver metastases and found a high CD8+/Foxp3+ ratio was an independent predictor of survival [22]. Specifically, the ratios of these subsets may provide a more comprehensive view of what occurs in the tumour microenvironment and which T cell subtype dominates or is likely to overshadow the functions of other T-cells. Previous works have demonstrated that high CD8 and Foxp3 expression contributed to better overall survival (OS) and disease-free survival (DFS) in HPSCC, yet the correlations of CD8/Foxp3 and CD8/CD4 wiht clinical outcomes remain unclear.

Based on the consideration that the quantitative ratios are probably more important in the tumour immune microenvironment, this study focuses on the prognostic significance of TILs and the relations of the CD8/Foxp3 and CD8/CD4 ratios with clinical outcomes and further seeks to determine more reliable biomarkers in a relatively larger advanced HPSCC cohort, which may appropriately select high-risk patients eligible for more aggressive therapeutic agents.

## Methods

### Specimens and0020patients

The present study enrolled 132 patients with HPSCC from 2013 to 2017, who underwent surgical treatment at the Eye and ENT Hospital of Fudan University, Shanghai, China. None of the patients received neoadjuvant chemotherapy or other therapies. All HPSCC specimens were fixed in 10% formalin and embedded in paraffin for histopathological analysis and immunohistochemistry. Haematoxylin-eosin (HE) staining of the sections was in an automated stainer/coverslipper workstation (HistoCore SPECTRA ST, Leica, Wetzlar, Germany). Complete clinical data were collected and all patients gave written informed consent before surgery. The Institutional Review Committee of the Eye and ENT Hospital granted ethical approval.

### Immunohistochemical (IHC) staining and evaluation

IHC staining was performed in automated immunostainer (Ventana Medical System, USA) using the following antibodies: anti-CD8 (SP16 Gene Tech, Shanghai, China, ready to use), anti-CD4 (EP204 Gene Tech, Shanghai, China, ready to use) and anti-Foxp3 (rabbit mAb, 98,377; CST, 1:200). Sections 4 μm were placed on glue-coated glass slides (PRO-01, Matsunami, Japan). Human tonsil sections were used as positive controls for CD8, Foxp3 and CD4. A negative control was performed by omitting the primary antibody. All conditions and procedures were defined as in our previous studies [23]. Tumoural parenchyma (tumour bed) was distinguished from the stroma using HE staining and the levels of CD8, Foxp3 and CD4 expression were counted manually under 10 randomly selected high-power fields (400X) for each slide. Areas of the tumour with haemorrhage or necrosis were avoided. Median values were used for cut-offs and the patient cohort was separated into high and low groups, as described in our previous study [23].

**Fig. 1:**
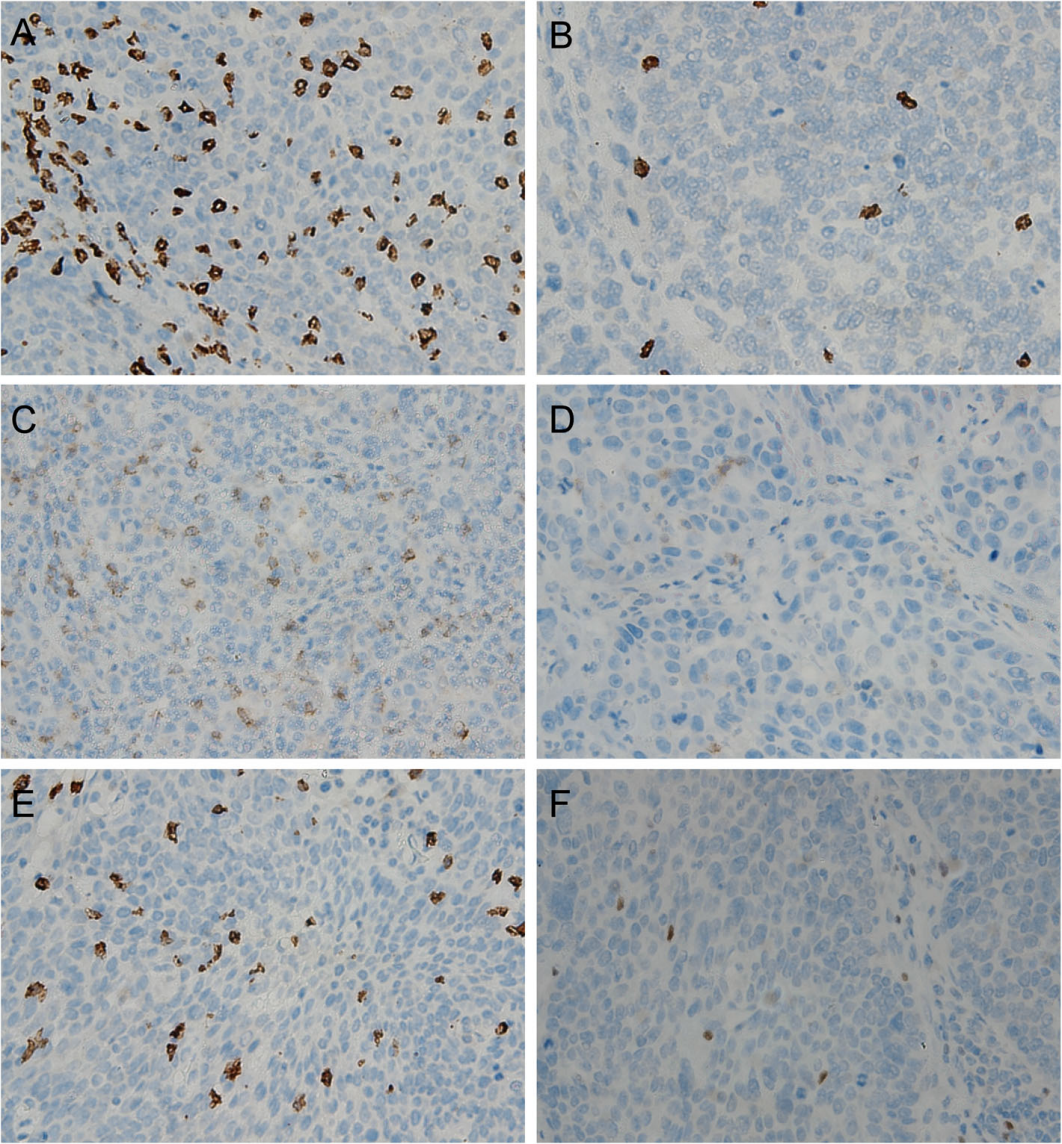
Immunohistochemical staining of CD8, CD4, and Foxp3 in the HPSCC cohort. **a** CD8^high^ and **b** CD8^low^ infiltration (200×); **c** CD4^high^ infiltration and **d** CD4^low^ infiltration (200×); **e** Foxp3^high^ infiltration and **f** Foxp3^low^ infiltration (200×). Abbreviations: HPSCC, hypopharyngeal squamous cell carcinoma

Representative images of the immunohistochemical detection of tumour-infiltrating T lymphocytes are shown in Fig. 1a-f. Two independent pathologists who were blinded to the patient data reviewed the slides. The medians were 80 for CD8 (range 1 to 900), 30 for Foxp3 (range 2 to 300) and 30 for CD4 (range 1to 400), respectively. We also investigated the ratios of CD8/Foxp3 and CD8/CD4, calculating them for each individual tumour. Similarly, the optimal cut-off points were calculated, along with their medians: the values were 2.50 (range 0.1 to 33.33) for CD8/Foxp3 and 3 (range 0.17 to 150) for CD8/CD4.

### Statistical analysis

Statistical analyses were performed by using SPSS (22.0, IBM, Armonk, NY, USA). Fisher’s exact test and the chi-squared test were used to evaluate the associations among the variables. The relationships between the different lymphocyte infiltrates were calculated using Pearson’s correlation coefficient. The Kaplan-Meier method and log-rank test were conducted to determine the prognosis at different survival end points. We used four clinical end points in this study: 1) overall survival (OS) was defined as the time from surgery until the date of death from any cause; 2) disease-free survival (DFS) was defined as the time from surgery until the date of the first recurrence/metastasis or death from any cause; 3) distant metastasis-free survival (DMFS) was defined as the time from surgery until the date of distant metastasis of the tumour or occurrence of death from any cause; and 4) local relapse-free survival (LRFS) was defined as the time from surgery until the date of local recurrence or death from any cause. Univariate and multivariate analyses (UVA and MVA) of prognostic factors were performed using the Cox proportional hazards model. The multivariate variables were adopted from their prognostic significance in UVA (*P* < 0.05). *P* < 0.05 was considered statistically significant.

## Results

### Patient characteristics

The study cohort included 132 patients in this who were diagnosed with HPSCC, and the clinical characteristics of these patients are summarized in Table 1. The samples included 131 males and 1 female with a median age of 60 years (range: 40–76 years). Twenty-nine (22%) patients had higher pathological grading (grade III), and 103 (78%) patients had lower pathological grading (grades I and II). As described above, the HPSCC patients were divided into 2 groups based on their overall staging group according to the AJCC 7th (American Joint Committee on Cancer) edition cancer staging system: namely overall staging group III (35, patients, 26.5%) and IVA or IVB (97 patients, 73.5%). Patients smoked at least 20 packs of cigarettes per year as many as 115 (87.1%) and smoking less than 20 packs group was 17 (12.9%). Regarding alcohol consumption, 28 (21.2%) patients consumed less than 10–40 g/day, and 104 (78.8%) patients consumed at least 40 g/day. Most tumours were located in the pyriform sinus (PS).

**Table 1:** Clinicopathological characteristics of 132 patients

Characteristic	N (%)
Age at diagnosis	
< 60y	71 (53.8)
≥ 60y	61 (46.2)
Sex	
Male	131 (99.2)
Female	1 (0.8)
Smoke history	
No	17 (12.9)
Yes	115 (87.1)
Drink history	
No	28 (21.2)
Yes	104 (78.8)
Site	
Pyriform sinus	116 (87.9)
Not pyriform sinus	16 (12.1)
Grade	
G1 + G2	103 (78.0)
G3	29 (22.0)
Stage	
III	35 (26.5)
IVA/IVB	97 (73.5)
T category	
T1–3	55 (41.7)
T4a	77 (58.3)
N category	
N0–1	55 (41.7)
N2–3	77 (58.3)
Laryngectomy	
Total	77 (58.3)
Partial	55 (41.7)

### Follow up

With a median follow-up of 28.4 months (interquartile range 20.9–39.1 months), the 3-year OS, DFS, DMFS and LRFS for the entire cohort were 68.2% (95% confidence interval [CI], 57.8 to 78.6%), 62.1% (95% CI, 52.1 to 72.1%), 72.6% (95% CI, 62.2 to 83.0%) and 79.7% (95% CI, 72.4 to 87.0%), respectively. During the follow-up period, 42 (31.8%) patients experienced treatment failure. A total of 16 (12.1%) and 17 (12.8%) patients had only locoregional recurrence or distant metastasis, respectively, and 9 (6.8%) patients had both.

### Association among different variables

**Fig. 2:**
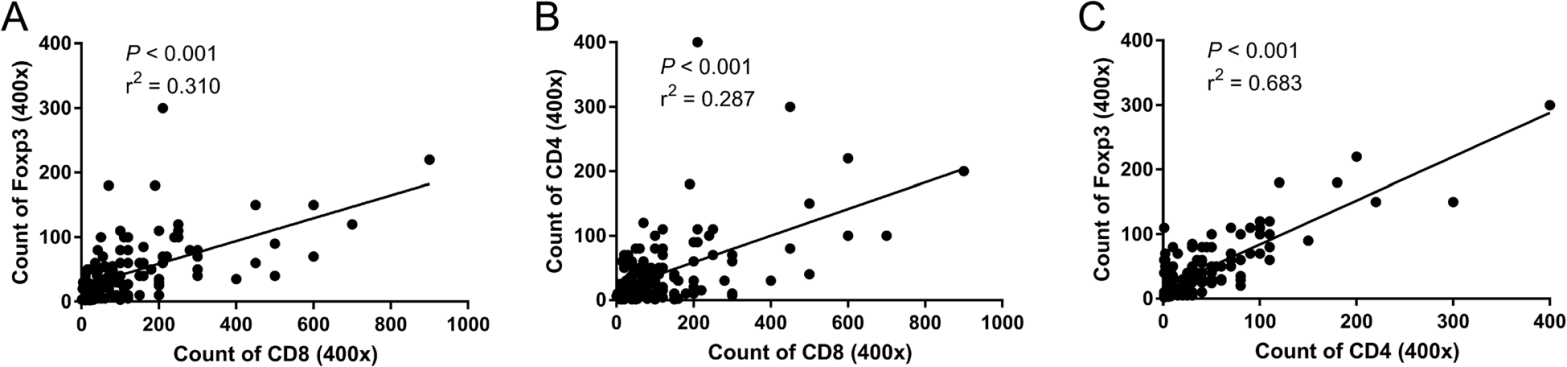
Correlations of the numbers of **a** CD8 and Foxp3, **b** CD8 and CD4, and **c** CD8 and Foxp3 infiltrating lymphocytes (*P* < 0.001)

Regarding the correlations of the immune markers with clinicopathological characteristics, high levels of TILs (CD8, Foxp3 and CD4) showed evident correlations with lower histopathological grade. The CD8/Foxp3 ratio was associated with the expression of CD8 and Foxp3, and the CD8/CD4 ratio correlated with each subtype of CD8 and CD4 infiltrates (*P* < 0.05). Similarly, Foxp3+ TILs exhibited an association with both overall staging group and T category (*P* = 0.048 and *P* = 0.046, respectively). We also found marked correlations among CD8, CD4 and Foxp3 using Pearson’s correlation coefficient (*P* < 0.001, Fig. 2a-c). Other relationships between immune marker expression and clinicopathological parameters are summarized in Table 2.

**Table 2:** Associations between the clinicopathological factors of HPSCC with the status of CD8, CD4, and Foxp3 infiltration and the CD8/Foxp3 and CD8/CD4 ratios (*N* = 132)

Characteristic	CD8		***P***	CD4		***P***	Foxp3		***P***	CD8/Foxp3		***P***	CD8/CD4		***P***
	low	high		low	high		low	high		low	high		low	high	
Age at diagnosis			0.033*			0.295			0.727			0.166			0.861
< 60y	31	40		31	40		37	34		33	38		34	37	
≥ 60y	38	23		33	28		29	32		36	25		31	30	
Sex			1.000			1.000			1.000			1.000			0.492
Male	1	0		0	1		0	1		1	0		1	0	
Female	68	63		64	67		66	65		68	63		64	67	
Smoke history			0.124			0.120			0.604			0.124			0.072
No	12	5		5	12		7	10		12	5		12	5	
Yes	57	58		59	56		59	56		57	58		53	62	
Drink history			0.671			0.834			0.832			1.000			0.832
No	16	12		13	15		13	15		15	13		13	15	
Yes	53	51		51	53		53	51		54	50		52	52	
Site			0.064			0.290			0.059			0.433			0.948
Pyriform sinus	57	59		54	62		54	62		59	57		57	59	
Not pyriform sinus	12	4		10	6		12	4		10	6		8	8	
Grade			0.003*			0.037*			0.003*			0.834			0.835
G1 + G2	61	42		55	48		59	44		53	50		50	53	
G3	8	21		9	20		7	22		16	13		15	14	
Stage			0.238			0.554			0.048*			0.609			1.000
III	15	20		15	20		12	23		17	18		17	18	
IVA/IVB	54	43		49	48		54	43		52	45		48	49	
T category			0.205			0.589			0.046*			0.449			0.718
T1–3	40	44		39	45		36	48		46	38		40	44	
T4a	29	19		25	23		30	18		23	25		25	23	
N category			1.000			0.599			0.289			0.536			0.860
N0–1	29	26		25	30		24	31		27	28		28	27	
N2–3	40	37		39	38		42	35		42	35		37	40	
Laryngectomy			0.379			0.599			0.480			0.725			0.295
Total	43	34		39	38		41	36		39	38		41	36	
Partial	26	29		25	30		25	30		30	25		24	31	
CD8 (cut off: 80)						0.001*			< 0.001*			0.001*			0.039*
Low				43	26		50	19		46	23		40	29	
High				21	42		16	47		23	40		25	38	
CD4 (cut off: 30)			0.001*						< 0.001*			0.163			< 0.001*
Low	43	21					49	15		29	35		15	49	
High	26	42					17	51		40	28		50	18	
Foxp3 (cut off: 30)			< 0.001*			< 0.001*						0.014*			
Low	50	16		49	17					27	39		29	37	0.296
High	19	47		15	51					42	24		36	30	

Abbreviations: *HPSCC* hypopharyngeal squamous cell carcinoma, *G1* Well differentiated, *G2* Moderately differentiated, *G3* Poorly differentiated

*The *P* value is significant

### Correlation with prognosis

**Fig. 3:**
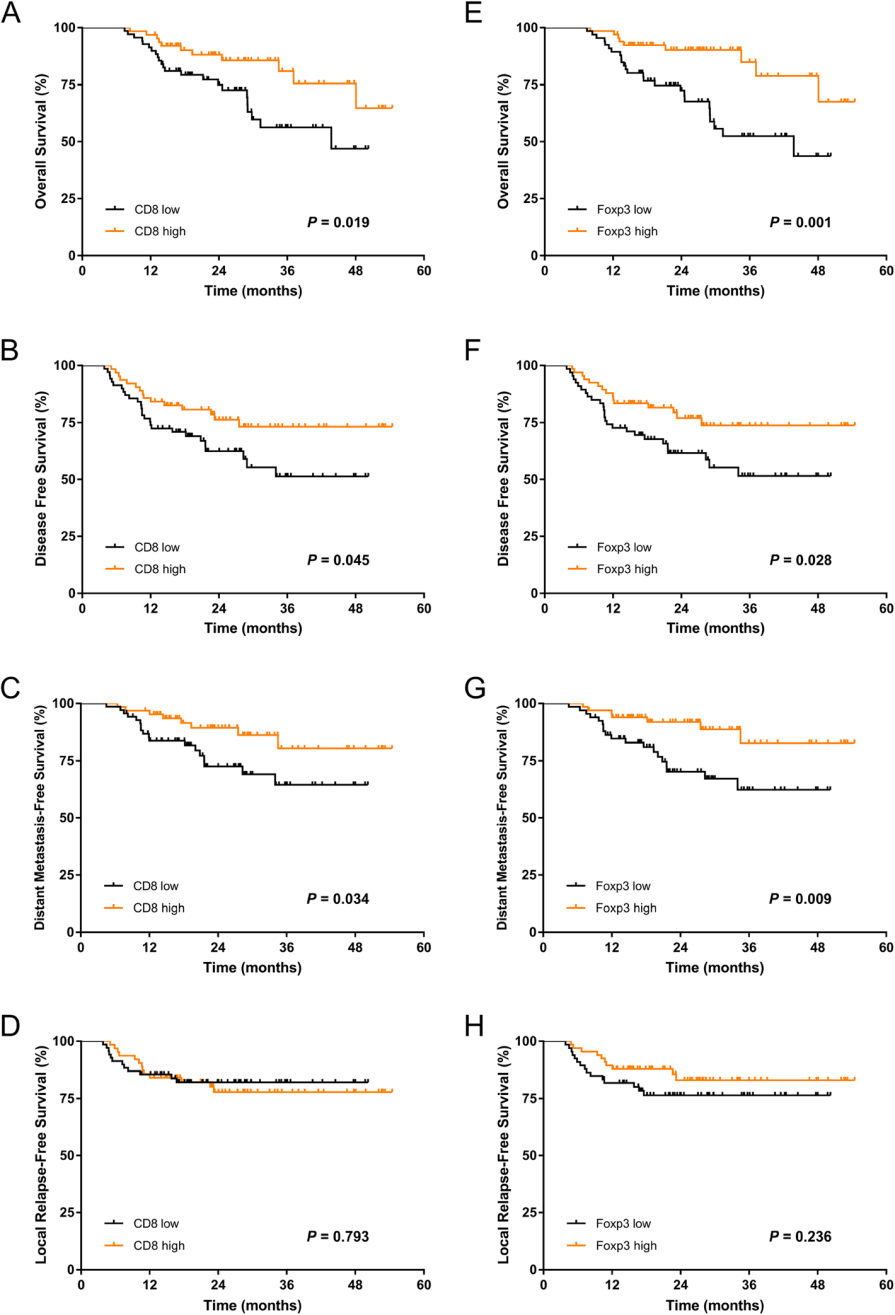
Kaplan-Meier curves of (**a**) overall survival, **b** disease-free survival, **c** distant metastasis-free survival, and **d** local relapse-free survival for patients stratified by high CD8 and low CD8 immune cell infiltration; **e** overall survival, **f** disease-free survival, **g** distant metastasis-free survival, and **h** local relapse-free survival for patients stratified by high and low Foxp3 immune cell infiltration. *P* values were calculated by the log-rank test

**Fig. 4:**
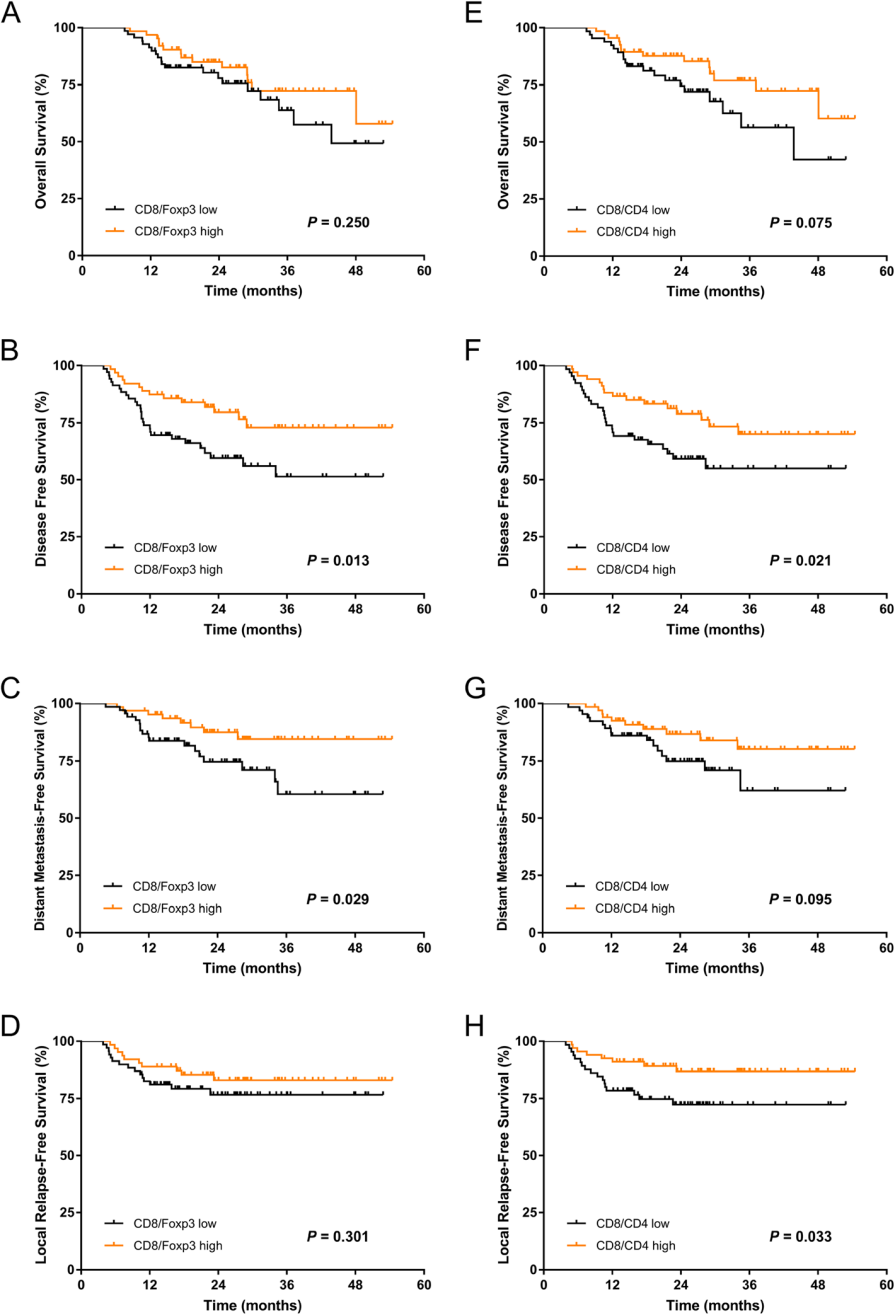
Kaplan-Meier curves of (**a**) overall survival, **b** disease-free survival, **c** distant metastasis-free survival, and **d** local relapse-free survival for patients stratified by high and low CD8/Foxp3 ratios; **e** overall survival, **f** disease-free survival, **g** distant metastasis-free survival, and **h** local relapse-free survival for patients stratified by high and low CD8/CD4 ratios. *P* values were calculated by the log-rank test

The Kaplan-Meier curves of 3-year OS, DFS, DMFS, LRFS for patients with TILs and the ratios are shown in Figs. 3-4. The 3-year OS, DFS, DMFS and LRFS rates according to high and low CD8 + TIL density, were 80.9% vs 56.3, 73.2% vs 51.4, 80.4% vs 64.5 and 77.8% vs 82.1%, respectively. Significant differences were found between the high and low CD8+ TIL groups in 3-year OS, DFS and DMFS but not in LRFS (Fig. 3a-d). Similarly, a higher Foxp3+ TIL level was also strongly correlated with better OS, DFS and DMFS (*P* = 0.001, *P* = 0.028 and *P* = 0.009, respectively, Fig. 3e-h). Further analysis revealed that patients with a high CD8/Foxp3 ratio had significantly better DFS and DMFS (*P* = 0.013 and *P* = 0.029, respectively) (Fig. 4b-c), while a higher CD8/CD4 ratio evidently improved 3-year DFS and LRFS compared with a lower CD8/CD4 ratio (*P* = 0.021 and *P* = 0.033, respectively) (Fig. 4f, h). In contrast, no associations were observed between the status of the CD8/Foxp3 ratio or the CD8/CD4 ratio and OS (Fig. 4a, e). Both UVA and MVA were performed to determine the associations between prognosis and clinicopathological variables (Table 3-4). The results revealed that a high ratio of CD8/Foxp3 remained an independent favourable prognostic factor for DFS (HR = 2.613; 95% CI, 1.203–5.673; *P* = 0.015) and DMFS (HR = 3.606; 95% CI, 1.334–9.748; *P* = 0.011). Furthermore, the CD8/CD4 ratio was also an independent prognostic factor for LRFS (HR = 2.418; 95% CI, 1.043–5.606; *P* = 0.040) in the MVA. In addition, Foxp3+ TIL, T category and site were found to be independent prognostic factors associated with DMFS, DFS and LRFS, respectively (Table 4).

**Table 3:** Univariate analyses of OS, DFS, DMFS and LRFS in the entire population (*N* = 132)

Variable	OS			DFS			DMFS			LRFS		
	HR	95%CI	***P***	HR	95%CI	***P***	HR	95%CI	***P***	HR	95%CI	***P***
Age, years (≥60y vs <60y)	0.521	0.254–1.073	0.077	0.637	0.341–1.188	0.156	0.787	0.361–1.715	0.547	0.594	0.263–1.346	0.212
Smoke (Yes vs No)	0.596	0.259–1.372	0.224	0.738	0.328–1.662	0.463	0.611	0.230–1.621	0.322	1.753	0.413–7.438	0.446
Drink history (Yes vs No)	0.748	0.348–1.607	0.457	0.945	0.452–1.976	0.881	0.822	0.330–2.050	0.675	1.991	0.596–6.653	0.263
Site (Not PS vs PS)	2.534	1.144–5.610	0.022*	2.524	1.206–5.284	0.014*	2.629	1.054–6.556	0.038*	2.600	1.038–6.513	0.041*
Grade (G3 vs G2 + G1)	0.689	0.285–1.665	0.408	1.033	0.508–2.102	0.929	1.283	0.539–3.053	0.573	1.586	0.684–3.675	0.282
Stage (IVA/IVB vs III)	1.785	0.739–4.313	0.198	2.457	1.035–5.834	0.042*	0.208	0.049–0.880	0.033*	2.848	0.852–9.518	0.089
T category (T4a vs T1–3)	2.259	1.151–4.436	0.018*	2.508	1.365–4.610	0.003*	2.681	1.231–5.842	0.013*	2.069	0.944–4.538	0.070
N category (N2–3 vs N0–1)	1.319	0.652–2.666	0.441	1.682	0.874–3.236	0.120	1.681	0.731–3.868	0.222	2.361	0.943–5.915	0.067
Laryngectomy (Total vs Partial)	1.896	0.853–4.215	0.117	1.860	0.951–3.638	0.070	2.233	0.894–5.578	0.085	2.370	0.946–5.938	0.066
CD8 (Low vs High)	2.324	1.127–4.795	0.022*	1.892	1.005–3.560	0.048*	2.391	1.038–5.505	0.040*	0.900	0.411–1.974	0.793
CD4 (Low vs High)	1.174	0.593–2.323	0.645	1.119	0.610–2.052	0.717	1.656	0.749–3.661	0.212	0.968	0.442–2.123	0.936
Foxp3 (Low vs High)	3.253	1.511–7.001	0.003*	1.999	1.063–3.760	0.032*	3.006	1.263–7.153	0.013*	1.615	0.726–3.596	0.240
CD8/Foxp3 (Low vs High)	1.490	0.752–2.953	0.253	2.205	1.159–4.195	0.016*	2.463	1.069–5.674	0.034*	1.522	0.683–3.391	0.305
CD8/CD4 (Low vs High)	1.857	0.930–3.705	0.079	2.058	1.099–3.851	0.024*	1.947	0.878–4.317	0.101	2.424	1.046–5.621	0.039*

Abbreviations: *PS* Pyriform sinus, *OS* Overall survival, *DFS* Disease-free survival, *DMFS* Distant metastasis-free survival, *LRFS* Local relapse-free survival, *G1* Well differentiated, *G2* Moderately differentiated, *G3* Poorly differentiated

*The *P* value is significant

**Table 4:** Multivariate analyses of OS, DFS, DMFS and LRFS in the entire population (*N* = 132)

Variable	OS			DFS			DMFS			LRFS		
	HR	95%CI	***P***	HR	95%CI	***P***	HR	95%CI	***P***	HR	95%CI	***P***
Site (Not PS vs PS)	1.621	0.706–3.726	0.255	1.756	0.810–3.807	0.154	1.549	0.614–3.909	0.354	2.590	1.034–6.491	0.042*
Stage (IVA/IVB vs III)				1.496	0.563–3.979	0.419	2.489	0.530–11.688	0.248			
T category (T4a vs T1–3)	1.880	0.947–3.735	0.071	2.196	1.077–4.476	0.030*	2.115	0.911–4.911	0.081			
CD8 (Low vs High)	1.231	0.509–2.977	0.644	0.716	0.300–1.709	0.452	0.698	0.217–2.242	0.546			
Foxp3 (Low vs High)	2.387	0.932–6.111	0.070	2.066	0.885–4.826	0.094	3.253	1.038–10.196	0.043*			
CD8/Foxp3 (Low vs High)				2.613	1.203–5.673	0.015*	3.606	1.334–9.748	0.011*			
CD8/CD4 (Low vs High)				1.708	0.871–3.349	0.119				2.418	1.043–5.606	0.040*

Multivariate cox regression analyses were performed for all variables that were significantly associated with survival in univariate analysis

Abbreviations: *PS* Pyriform sinus, *OS* Overall survival, *DFS* Disease-free survival, *DMFS* distant metastasis-free survival, *LRFS* Local relapse-free survival, *G1* Well differentiated, *G2* Moderately differentiated, *G3* poorly differentiated

*The *P* value is significant

## Discussion

Our study is the first to evaluate lymphocyte ratios in advanced HPSCC and their correlations with clinicopathological characteristics and prognosis in more than 100 patients who underwent surgery. The results indicated that high ratio of CD8/Foxp3 accurately predicted improved prognosis with better DFS and DMFS, and increased CD8/CD4 ratio was a markedly indicator of improved LRFS. Although Foxp3+ TILs were an independent prognostic factor for DMFS, we could not demonstrate any significant association between CD8+ TIL expression and clinical outcomes in MVA.

In recent years, it has become clear that assessing immune infiltration is of greater prognostic significance in a variety of tumours [15]. CD8+ CTLs are directly capable of killing tumour cells and positively affect prognosis in a broad range of tumour types, including breast cancer, ovarian cancer, head and neck cancer and lung cancer [24–27]. In accordance with previous results, we demonstrated that higher CD8+ infiltration is associated with longer OS, DFS and DMFS in UVA. However, several other studies indicated that there is no such correlation with prognosis. One study even found a negative effect of CD8+ TILs on survival, but this did not reach statistical significance in multivariate analysis [28–30]. In contrast, as one of the paradoxically functional components of the tumour-related immune system, Foxp3+ TILs are considered to be the most specific Treg marker involved in maintaining immune tolerance to the host. In tumour progression, Tregs produce the inhibitory cytokines interleukin 10, transforming growth factor β and haemoglobin oxygenase 1 to achieve immune escape [31]. Therefore, many studies have suggested that higher Foxp3 Treg infiltration is associated with poor prognosis in various malignancies including breast, lung, cervical, oral cavity and ovarian cancers [32, 33]. On the other hand, accumulating evidence has emerged that in other cancers, including HPSCC, their presence was associated with better prognosis [23, 34–36]. To date, the role of Foxp3 regulator T cells in cancer is still conflicting. Assessing cytotoxic CD8+ T cells and regulatory Foxp3 T cells together, as the two major components of the tumour-related immune system, could provide more precise estimates of their effects on HPSCC patient survival. The present study also demonstrated that higher Foxp3 TIL density in UVA led to significantly better OS, DFS and DMFS outcomes, but only DMFS had independent prognostic significance in MVA, which is slightly different from the findings of our previous study [23]. Furthermore, the current data showed that CD4 TIL density had no impact on survival but showed strong correlations with CD8 and Foxp3. We assumed that the presence of CD4 T cells alone is not associated with prognosis and that these cells may interact with other subsets, exerting many more effects in the tumour microenvironment.

We measured the relative number of TILs and explored the association between different subsets, and the data indicated positive correlations among CD8, Foxp3 and CD4 T cells. As an indicator of the balance between CD8+ TILs and Foxp3 Tregs in the tumour microenvironment, the CD8/Foxp3 ratio appeared to be useful for predicting clinical outcomes. In reviewing the literature, we found that the CD8/Foxp3 ratio had a positive effect on prognosis in a number of tumours, including osteosarcoma, colorectal cancer and breast cancer [21, 33, 37–39]. For patients with tonsillar cancer, a high CD8/Foxp3+ ratio positively correlated with DFS [40]. Ni et al. reported that increased CD8/Foxp3 ratios were associated with improved OS, DFS and tumour stage in tongue cancer but were not an independent prognostic factor in MVA [28]. Similar to these studies, this cohort demonstrated that a high CD8/Foxp3 ratio correlated with favourable prognosis and CD8 expression, which further confirmed that CD8+ TILs are associated with good prognosis in advanced HPSCC patients. Additionally, when using different survival end points, the CD8/Foxp3 ratio consistently served as an independent prognostic factor for DFS and DMFS in MVA. A large meta-analysis of TIL phenotyping, encompassing 33 studies and nearly 10,000 patients, indicated that lymphocyte ratios, particularly the CD8/Foxp3 ratio, have more prognostic potential than individual lymphocytic subtypes [31]. Although an investigation showed no significant correlation between the CD8/Foxp3 ratio and survival in ovarian cancer [41], the CD8/Foxp3 ratio was found to be a promising prognostic marker in advanced HPSCC. Additionally, the current study also identified that a high CD8/CD4 ratio was associated with better DMFS and LRFS in UVA and was an independent prognostic factor for LRFS in MVA, although CD4 infiltrating T cells alone were not significantly correlated with survival implications. Consistent with our results, previous studies have reported that a high stromal CD8/CD4 ratio was found to be an independent favourable prognostic factor in oral squamous cell carcinoma, and one study revealed that the CD8/CD4 ratio was higher in cases without metastasis and in low-grade lesions [42, 43]. Moreover, studies in lung cancer suggested that the CD8/CD4 ratio in patients in the non-metastasis group was remarkably higher, and these patients had a significantly better overall survival rate than patients with a low CD8/CD4 ratio [44, 45]. Other studies of different lesions also observed that higher CD8/CD4 ratios were associated with improved outcome [46, 47]. In addition, high CD8/CD4 was associated with improved short-term survival in head and neck squamous cell carcinoma and was significantly correlated with the absence of lymph node metastases in cervical carcinomas, thus indicating a favourable prognosis [48, 49]. However, there were also instances in which CD8/CD4 ratio was not linked to clinical outcomes, and some researchers even reported that a high CD8/CD4 ratio was associated with alcohol use and poor tumour differentiation [50]. In general, the tendency for better clinical outcomes of patients with a high CD8/CD4 ratio is notable in HPSCC, despite the prognostic significance being very different from that in other tumours. It is also noteworthy that the current study found Foxp3 to be an independent prognostic factor for DMFS, whereas CD8 did not show any significance in MVA. These results were different from those of previous study because lymphocyte ratios were included in the MVA of this cohort, which again supported the idea that the CD8/Foxp3 and CD8/CD4 ratios were more indicative of prognosis than each subtype alone. Altogether, these findings confirm that not only the infiltration density of TIL subsets but also the ratios of TILs, particularly the CD8/Foxp3 and CD8/CD4 ratios, have important impacts on patient outcomes and could potentially be taken into account when considering patient prognostication and treatment stratification. In the era of immunotherapy, these immune biomarkers may provide new clues to therapeutic strategies and are speculated to be possible predictive markers of treatment efficacy. Further studies are needed to validate the results of the present study in a large cohort with neoadjuvant settings.

Although the present results are very promising, there are some limitations. First, all the markers in this study are for the advanced clinical stage of HPSCC (III and IVA), and early clinical stage (I-II) cases are also needed for further testing. Second, a short follow-up resulted in a limited number of patients with locoregional recurrence and distant metastasis. Third, this study cohort consisted of 131 males and 1 female who underwent surgical treatment because most female patients chose laryngeal preservation treatment. Further investigations are warranted to overcome these limitations as much as possible.

## Conclusion

This study demonstrated that high TIL levels are of prognostic significance in HPSCC, while the ratios between these subsets may be more informative. We stressed that a high ratio of CD8/Foxp3 accurately predicted prognosis for improved DFS and DMFS, and an increased CD4/CD8 ratio was an independent prognostic factor for better LRFS. These findings will improve our understanding of the clinical significance of immune cells in HPSCC.

## Data Availability

The datasets used and analysed during the current study are available from the corresponding author on reasonable request.

## Abbreviations

HPSCC: Hypopharyngeal squamous cell carcinoma
CCRT: Concurrent chemoradiation therapy
TILs: Tumour-infiltrating lymphocytes
CTLs: Cytotoxic T lymphocytes
NSCLC: Non-small cell lung cancer
Tregs: Regulatory T cells
HE: Haematoxylin-eosin
IHC: Immunohistochemical
OS: Overall survival
DFS: Disease-free survival
DMFS: Distant metastasis-free survival
LRFS: Local relapse-free survival
MVA: Multivariate analysis
UVA: Univariate analysis
AJCC: American Joint Committee on Cancer
PS: Pyriform sinus
